# Early outcomes of oblique lateral interbody fusion with posterior fixation versus posterior interbody fusion with fixation for treating adult degenerative scoliosis

**DOI:** 10.1186/s13018-023-04363-7

**Published:** 2023-11-17

**Authors:** Xiangyu Li, Xiaolong Chen, Yu Wang, Ashish D. Diwan, Shibao Lu

**Affiliations:** 1 Department of Orthopedics, Xuanwu Hospital, Capital Medical University, No.45 Changchun Street, Xicheng District, Beijing, China; 2 National Clinical Research Center for Geriatric Diseases, Beijing, China; 3 St. George and Sutherland Clinical School, University of New South Wales, Level 3, WR Pitney Building, Kogarah, Sydney, NSW 2217 Australia; 4 Spine Service, Department of Orthopaedic Surgery, St. George Hospital Campus, Sydney, NSW Australia

**Keywords:** Oblique lateral interbody fusion, Posterior interbody fusion, Adult degenerative scoliosis, Surgical trauma

## Abstract

**Objective:**

To compare the surgical trauma and outcomes between oblique lateral interbody fusion (OLIF) and posterior fixation and posterior lumbar interbody fusion (PLIF) with fixation for adult degenerative scoliosis (ADS).

**Methods:**

We included ADS patients who underwent OLIF with fixation or PLIF with fixation treatment from June 2020 to December 2022. The preoperative and postoperative spinal pelvic parameters were measured using X-rays. Clinical symptoms were measured using the Oswestry Disability Index and a visual analog scale. We recorded operation time, intraoperative blood loss, blood transfusion, albumin infusion, surgical fixation segment, surgical osteotomy segment, time, and drainage volume.

**Results:**

Forty patients with ADS were included: 20 with OLIF with posterior fixation and 20 with PLIF matched for age, sex, pelvic incidence, and Cobb angle with the OLIF group. There were no significant differences in age, gender, BMI, preoperative spinal parameters, or preoperative clinical symptoms between the groups (*p* > 0.05). There were no statistical differences in postoperative spinal parameters or clinical symptoms (*p* > 0.05). Patients in the OLIF group had less intraoperative blood loss (*p* < 0.01) and fewer intraoperative blood transfusions (*p* < 0.001) than the posterior surgery group. The number of fixed segments was fewer (*p* < 0.01), and there were fewer total osteotomy segments (*p* < 0.001).

**Conclusion:**

OLIF with posterior fixation surgery can achieve the same corrective effect and efficacy as a posterior internal fusion with fixation surgery for treating ADS. OLIF with posterior fixation surgery causes less trauma and reduces the number of fixation segments.

## Introduction

Adult degenerative scoliosis (ADS) is a spinal deformity with a scoliotic angle of over 10 degrees that develops post-adulthood. ADS usually begins around age 50 and progresses with aging [1, 2]. The presentations of ADS include back pain, radiating pain, neurological symptoms, and deformity. Surgery should be considered for patients with severe symptoms and failure of conservative treatment [2, 3].

The posterior approach of long fusion with osteotomy correction of the deformity is the conventional technique to treat ADS [2]. However, iatrogenic paraspinal muscle injury, significant blood loss during surgery, and long-segment fixation hinder early and rapid rehabilitation. Reducing surgical trauma is a perennial problem.

Besides posterior approach, the approaches to the spine forward of the vertebral canal, such as oblique lateral interbody fusion (OLIF), lateral lumbar interbody fusion (LLIF), and extreme lateral interbody fusion (XLIF), are increasingly used in performing fusion [4]. Studies had confirmed that LLIF was effective for ADS with less trauma compared conventional to posterior approach [5, 6]. XLIF, as a minimally invasive surgery, also reduced number of levels of lumbar scoliosis [7]. Thus, the application of anterior lumbar fusion in ADS has broad prospects.

Oblique lateral interbody fusion (OLIF) is gaining popularity because it provides indirect decompression, preserves the posterior column structure, and causes less trauma and blood loss [8–10]. Some studies have reported OLIF used in ADS [11–13]. By inserting large cages, OLIF achieves vertebral body distraction, disk height restoration, and lumbar lordosis remodeling.

Compared with posterior correction and fixation, the affection of OLIF on the second-stage posterior surgery osteotomy level, the number of fusion segments, and the operation time for treating the patients with ADS are still unknown. Therefore, we performed a cohort study to compare the clinical and radiological outcomes between OLIF and posterior lumbar interbody fusion (PLIF) for treating patients with ADS.

## Materials and methods

### Patient selection

In this retrospective study, we obtained ethical approval from the ethics committee at our hospital. A total of 101 patients underwent OLIF with posterior fixation, and 236 patients underwent three (or more)-level PLIF by a single surgeon from June 2020 to December 2022. All patients meet the following surgical indications: (1) recurrent low back and leg pain gradually worsens, seriously affecting normal life and ineffective non-surgical treatment; (2) accompanying lumbar spinal stenosis or stubborn nerve root pain and neurological dysfunction, with intermittent claudication; (3) muscle strain secondary to imbalance of spine or scoliosis; (4) progressive exacerbation of scoliosis, with scoliosis progression > 10°; (5) pulmonary dysfunction secondary to spinal deformities, affecting normal daily life; and (6) there were no obvious surgical contraindications during preoperative evaluation. The goal of surgery is to relieve the nerve root compression and correct deformity [2].

Inclusion criteria are as follows: (1) patients with more than 10 degrees of Cobb angle; (2) aged more than 40 years old; and (3) a completion of more than 3 months follow-up.

Exclusion criteria were as follows: (1) idiopathic or congenital scoliosis; (2) history of previous spine surgery; (3) severe osteoporosis (*T*-score < − 3.5); (4) spinal deformity caused by spinal tumor or infection; and (5) paraplegia or difficulty standing.

The following data were collected: age, sex, body mass index (BMI), duration of follow-up time period, and major intraoperative and perioperative complications. Albumin and hemoglobin were recorded before and on the first day after surgery.

### Surgical procedures

All patients underwent general anesthesia before surgery. In the OLIF group, the procedure used a lateral approach. The incision was transverse, centered on the anterior margin of the disk space. The natural space between the retroperitoneal abdominal aorta and the front edge of the psoas major muscle was used to remove the intervertebral disk. PEEK intervertebral cages filled with artificial bone were inserted into intervertebral space. Subsequently, or in the second stage, patients were placed in the prone position and underwent posterior fixation. Posterior decompression was performed according to compression segments. Pedicle screw augmentation with cement or proximal segment strengthening was performed according to the bone condition.

In the PLIF group, the patients were positioned prone. An incision was made in the middle of the posterior square of the back. Paraspinal muscles were dissected layer by layer to expose the lamina and articular process. Decompression was performed according to compression segments. Fusion was performed with cages filled with autograft from a laminectomy. Fixation was performed with rods and pedicle screws. Pedicle screw augmentation with cement or proximal segment strengthening was performed according to the bone condition.

The posterior osteotomies were performed according to the stiff deformity and imbalance to correct the deformity and rebalance the spine. The osteotomies were usually Smith–Petersen osteotomy (grade I), Ponte osteotomy (grade II), and pedicle subtraction osteotomy (grade III). Operation time, fixation segments, osteotomy level, blood loss, blood transfusion (autologous and allogeneic blood), human albumin 20% infusion, time, and drainage volume were recorded.

### Imaging assessment

**Fig. 1:**
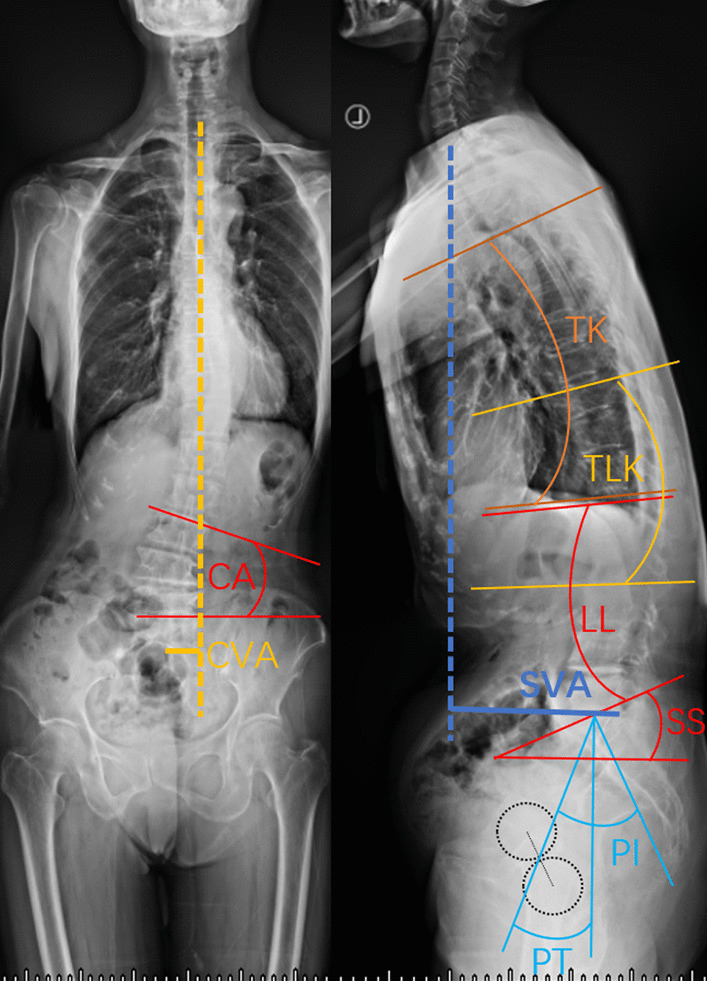
Method to measure spine parameters. PI, pelvic incidence; PT, pelvic tilt; SS, sacral slope; LL, lumbar lordosis; TK, thoracic kyphosis; TLK, thoracolumbar kyphosis; SVA, sagittal vertical axis; CA, Cobb angle; CVA, coronal vertical axis

Whole-spine standing radiographs were obtained before surgery and at follow-up in a standardized upright position. Spine alignment measurements in this study were defined with neutral standing X-ray images (Fig. 1).

Pelvic tilt (PT) was defined by the angle between the line connecting the midpoint of the S1-endplate to the axis of the femoral heads and the vertical plane. The sacral slope (SS) was defined as the angle between the horizontal and sacral endplates. Pelvic incidence (PI) was defined as the angle perpendicular to the sacral endplate at its midpoint and the line connecting this point to the axes of the femoral heads. Thoracic kyphosis (TK) was measured from the upper endplate of T4 to the lower endplate of T12. Lumbar lordosis (LL) was defined as the angle between S1-endplate and L1 upper endplate. Thoracolumbar kyphosis (TLK) was measured by the CA between the upper endplate of T10 and the lower endplate of L2. The C7 sagittal vertical axis (SVA) was defined by the horizontal distance from the superior posterior end of the upper sacral endplate to the C7 plumbline. The C7 coronal vertical axis (CVA) is the distance from the C7 plumb line to the central sacral vertical line. The scoliosis Cobb angle (CA) was also measured. We defined lordosis as a positive value and kyphosis as a negative value.

### Clinical assessment

The lumbar function was evaluated using the Oswestry Disability Index (ODI), and the degree of low back pain and lower limb pain was evaluated using a visual analog scale (VAS).

### Data analysis

All collected data were analyzed using IBM SPSS Statistics, version 25.0 (IBM Corp., Armonk, NY, USA). One-to-one propensity score matching was performed. Covariates were age, sex, PI, and CA. OLIF cases were matched in a 1:1 ratio to PLIF patients based on the propensity score with a standard caliper width of 0.2. Statistical analysis was performed using the *T*-test for parametric test or Wilcoxon test for nonparametric test. The results were expressed as the mean value ± standard deviation. A probability (*p*) value of < 0.05 was considered statistically significant.

## Results

### Demographics

In the OLIF group, 20 of 101 patients underwent OLIF for ADS. Twenty of 236 patients in the PLIF group were matched with 20 patients in the OLIF group using a match tolerance of 0.2 based on age, sex, PI, and CA. The average age was 70.45 ± 8.94 years old in the OLIF group and 70.55 ± 6.93 years old in the PLIF group. There were no significant differences in age, gender, BMI, or follow-up (*p* > 0.05, Table 1).

**Table 1:** Characteristics

Characteristics	PLIF (20)	OLIF (20)	*p* value
Age (years)	70.55 ± 6.93	70.45 ± 8.94	0.969
Gender (male/female)	13/7	14/6	0.739
BMI (kg/m^2^)	24.73 ± 2.65	23.67 ± 3.90	0.323
Follow-up (months)	15.15 ± 5.89	14.90 ± 5.88	0.894

BMI, body mass index

### Preoperative characteristics

The radiological parameters before surgery are displayed in Table 2. There were no significant differences in preoperative PI, PT, SS, LL, TLK, TK, SVA, CA, or CVA between the two groups (*p* = 0.841, *p* = 0.168, *p* = 0.128, *p* = 0.256, *p* = 0.196, *p* = 0.848, *p* = 0.187, *p* = 0.952, and *p* = 0.674, respectively). Hemoglobin and albumin did not differ between the two groups (*p* = 0.414 and *p* = 0.105, respectively). The mean ODI score was 32.25 ± 8.06% in the PLIF group and 30.95 ± 10.66% in the OLIF group. The mean lumbar VAS score was 5.80 ± 1.52 in the PLIF group and 5.85 ± 1.42 in the OLIF group. The mean leg VAS score was 3.25 ± 1.65 in the PLIF group and 3.20 ± 1.91 in the OLIF group.

**Table 2:** Preoperative parameters

Preoperative parameters	PLIF (20)	OLIF (20)	*p* value
PI (°)	48.42 ± 10.91	47.67 ± 8.46	0.841
PT (°)	29.49 ± 12.56	24.59 ± 9.26	0.168
SS (°)	18.83 ± 8.69	23.09 ± 8.64	0.128
LL (°)	21.01 ± 14.87	26.16 ± 13.32	0.256
TLK (°)	− 19.42 ± 17.87	− 12.73 ± 14.03	0.196
TK (°)	− 25.69 ± 15.98	− 24.73 ± 15.72	0.848
SVA (cm)	7.75 ± 5.86	5.55 ± 4.37	0.187
CA (°)	25.60 ± 10.78	25.81 ± 11.15	0.952
CVA (cm)	2.24 ± 1.38	2.03 ± 1.73	0.674
Hemoglobin (g/L)	128.90 ± 10.86	125.25 ± 16.53	0.414
Albumin (g/L)	37.64 ± 3.08	39.35 ± 3.40	0.105
ODI (%)	32.25 ± 8.06	30.95 ± 10.66	0.666
VAS of back	5.80 ± 1.52	5.85 ± 1.42	0.915
VAS of leg	3.25 ± 1.65	3.20 ± 1.91	0.930

PLIF, posterior lumbar interbody fusion; OLIF, oblique lateral interbody fusion; PI, pelvic incidence; PT, pelvic tilt; SS, sacral slope; LL, lumbar lordosis; TK, thoracic kyphosis; TLK, thoracolumbar kyphosis; SVA, sagittal vertical axis; CA, Cobb angle; CVA, coronal vertical axis; ODI, Oswestry Disability Index; VAS, visual analog scale

### Surgical characteristics

Although 13 patients in OLIF group had second-stage surgeries for posterior fixation, PLIF and OLIF demonstrated similar total operation times (*P* = 0.055). The OLIF group showed less blood loss (*P* = 0.006), fewer intraoperative blood transfusions (*p* < 0.001), fewer postoperative blood transfusion (*p* = 0.020), fewer fixation segments (*p* = 0.003), and fewer osteotomy levels (*p* < 0.001). The OLIF group had less drainage time and volume (*p* = 0.010, *p* = 0.003). The groups had similar interbody fusion levels, postoperative albumin infusion, postoperative hemoglobin, and postoperative albumin (*p* = 0.374, *p* = 0.090, *p* = 0.498, and *p* = 351, respectively) (Table 3).

**Table 3:** Surgical characteristics and trauma

	PLIF (20)	OLIF (20)	*p* value
Operation time (h)	6.88 ± 1.22	5.75 ± 2.22	0.055
Second-stage surgeries (*n*)	0	13	< 0.001
Blood loss (ml)	1067.50 ± 712.72	475.00 ± 559.60	0.006
Intraoperative blood transfusion (ml)	1175.10 ± 805.67	274.65 ± 443.18	< 0.001
Fixation segments (*n*)	8.35 ± 2.58	5.35 ± 3.30	0.003
Interbody fusion levels (*n*)	2.60 ± 1.05	2.85 ± 0.67	0.374
Total osteotomy levels (*n*)	83	35	< 0.001
Grade I (*n*)	9	6	
Grade II (*n*)	69	28	
Grade III (*n*)	5	1	
Postoperative blood transfusion (ml)	410.00 ± 516.98	95.00 ± 232.78	0.020
Postoperative albumin infusion (g)	11.00 ± 18.89	3.00 ± 7.33	0.090
Postoperative hemoglobin (g/L)	113.65 ± 16.60	110.15 ± 16.60	0.498
Postoperative albumin (g/L)	29.39 ± 4.31	30.75 ± 4.76	0.351
Drainage time (days)	6.60 ± 1.39	4.50 ± 3.19	0.010
Drainage volume (ml)	1384.55 ± 483.48	774.65 ± 723.46	0.003

PLIF, posterior lumbar interbody fusion; OLIF, oblique lateral interbody fusion

### Radiographic and clinical outcomes

We compared the correction ability between the groups. Changes in LL, PT, SS, TK, CA, and SVA were similar between the groups (*p* = 0.566, *p* = 0.503, *p* = 0.241, *p* = 0.842, *p* = 0.343, and *p* = 0.656, respectively) (Table 4).

**Table 4:** Radiographic parameter changes

Change	PLIF (20)	OLIF (20)	*p* value
LL (°)	14.44 ± 12.04	12.08 ± 13.69	0.566
PT (°)	− 7.74 ± 9.52	− 5.85 ± 8.10	0.503
SS (°)	8.40 ± 9.11	5.07 ± 8.50	0.241
TK (°)	− 3.48 ± 9.57	− 2.80 ± 11.73	0.842
CA (°)	− 14.29 ± 9.70	− 17.44 ± 9.70	0.343
SVA (cm)	− 4.18 ± 5.60	− 3.50 ± 3.78	0.656

PLIF, posterior lumbar interbody fusion; OLIF, oblique lateral interbody fusion; PT, pelvic tilt; SS, sacral slope; LL, lumbar lordosis; TK, thoracic kyphosis; SVA, sagittal vertical axis; CA, Cobb angle

We compared the radiological parameters and clinical outcomes at follow-up between the groups (Table 5). There were no differences in postoperative PI, PT, SS, LL, TLK, TK, SVA, CA, and CVA between the groups (*p* = 0.427, *p* = 0.162, *p* = 0.659, *p* = 0.326, *p* = 0.056, *p* = 0.584, *p* = 0.167, *p* = 0.085, and *p* = 0.820, respectively). The mean ODI, lumbar VAS, and leg VAS at the final follow-up were significantly lower than the preoperative values in both groups (*p* < 0.001). No significant differences were found in postoperative ODI, VAS of the back, or VAS of the leg (*p* = 0.217, *p* = 0.092, *p* = 0.856, respectively).

**Table 5:** Radiological parameters and clinical outcomes at follow-up

Postoperative parameters	PLIF (20)	OLIF (20)	*p* value
PI (°)	48.97 ± 8.97	46.90 ± 7.25	0.427
PT (°)	21.75 ± 7.91	18.74 ± 5.15	0.162
SS (°)	27.22 ± 7.08	28.16 ± 6.23	0.659
LL (°)	35.44 ± 9.70	38.23 ± 9.70	0.326
TLK (°)	− 6.10 ± 6.13	− 10.15 ± 6.83	0.056
TK (°)	− 29.16 ± 10.18	− 27.52 ± 8.61	0.584
SVA (cm)	3.57 ± 4.10	2.05 ± 2.56	0.167
CA (°)	11.31 ± 6.07	8.37 ± 4.30	0.085
CVA (cm)	1.64 ± 1.14	1.79 ± 2.77	0.820
ODI (%)	14.20 ± 6.73	11.65 ± 6.09	0.217
VAS of back	3.25 ± 1.65	3.20 ± 1.91	0.092
VAS of leg	0.85 ± 0.81	0.90 ± 0.91	0.856

PLIF, posterior lumbar interbody fusion; OLIF, oblique lateral interbody fusion; PI, pelvic incidence; PT, pelvic tilt; SS, sacral slope; LL, lumbar lordosis; TK, thoracic kyphosis; TLK, thoracolumbar kyphosis; SVA, sagittal vertical axis; CA, Cobb angle; CVA, coronal vertical axis; ODI, Oswestry Disability Index; VAS, visual analog scale

### Complications

Three case of urinary tract infection, two cases of dural tears, one case of cage subsidence, one case of local hematoma, one case of deep venous thrombosis (DVT), and one case of deep wound infection were confirmed in PLIF group. The most common intraoperative complication was endplate injury (3/20) in the OLIF group. The postoperative complications included transient psoas (6/20), cage subsidence (5/20), delirium (1/20), and DVT (1/20) in OLIF group. No major complication that required prolonged hospitalization or revision was found in both two groups.

## Discussion

ADS is associated with sagittal and coronal plane malalignment caused by asymmetric intervertebral disk degeneration and facet joint degeneration [14]. The presentations of ADS include back pain, radiating pain, neurological symptoms, and deformity. Adequate decompression and deformity correction are the goal of ADS surgery for patient satisfaction and health-related quality of life improvement [15]. Posterior spinal osteotomies and fixation have been used to treat ADS for many years. However, PLIF with osteotomies usually introduces more surgical trauma, including more intraoperative blood loss, more grade and level of osteotomies, and longer drainage time, than with OLIF.

The OLIF technique involves inserting a very large cage with the anterior approach, which can directly increase the height of the interbody, indirectly decompress the spinal canal, and improve coronal and sagittal spinal alignment [8, 9]. Studies showed that OLIF is a safe and effective method for treating ADS [13, 16]; nevertheless, it remained unclear whether OLIF with posterior fixation required more time or introduced less operative trauma than PLIF alone. Therefore, we compared the clinical and radiological outcomes between OLIF and PLIF when treating ADS.

### Clinical outcomes

We found that both groups' VAS-back, VAS-leg, and ODI scores improved significantly. The groups had no differences in postoperative VAS-back, VAS-leg, or ODI scores. These findings suggest OLIF can achieve clinical outcomes that are not inferior to those of PLIF. Shimizu et al. reported that OLIF had the same short-term efficacy as PLIF in treating lumbar spinal stenosis [8]. Zhao et al. demonstrated the efficacy of OLIF with anterolateral single screw-rod fixation in lumbar degenerative disk disease [17]. Zhu et al. reviewed 16 studies and found that OLIF was effective for symptom relief [13]. Therefore, we hypothesized that OLIF with fixation would provide good outcomes based on repeated decompression and deformity improvement.

### Deformity correction

There was a reduced coronal CA postoperatively in both groups, 14.29 ± 9.70° CA reduction in the PLIF group and 17.44 ± 9.70° in the OLIF group. There was no difference in the correction of coronal CA. The wide cages in the OLIF group provided the ability to correct CA. Many studies found that sagittal imbalance is associated with quality of life; thus, restoring sagittal balance can improve outcomes [1, 3, 18, 19]. Studies have confirmed the abilities of OLIF in restoration of disk height and correction of lumbar lordosis angle [20–22]. The huge cages with 6° or 12° radian provided ability to correct lumbar lordosis angle. The correction abilities of sagittal deformity, including LL, PT, SS, and SVA corrections, were similar between the groups. PLIF relies on ‘shortening the spine’ for corrective purposes, while OLIF achieves corrective purposes by ‘extending the spine’. These findings suggest that OLIF is effective for the sagittal and coronal planes correction in ADS. Studies also reported that LLIF and XLIF had the ability of CA correction, which verified the role of intervertebral space expansion in restoring deformities [23, 24].

### Operative trauma

**Fig. 2:**
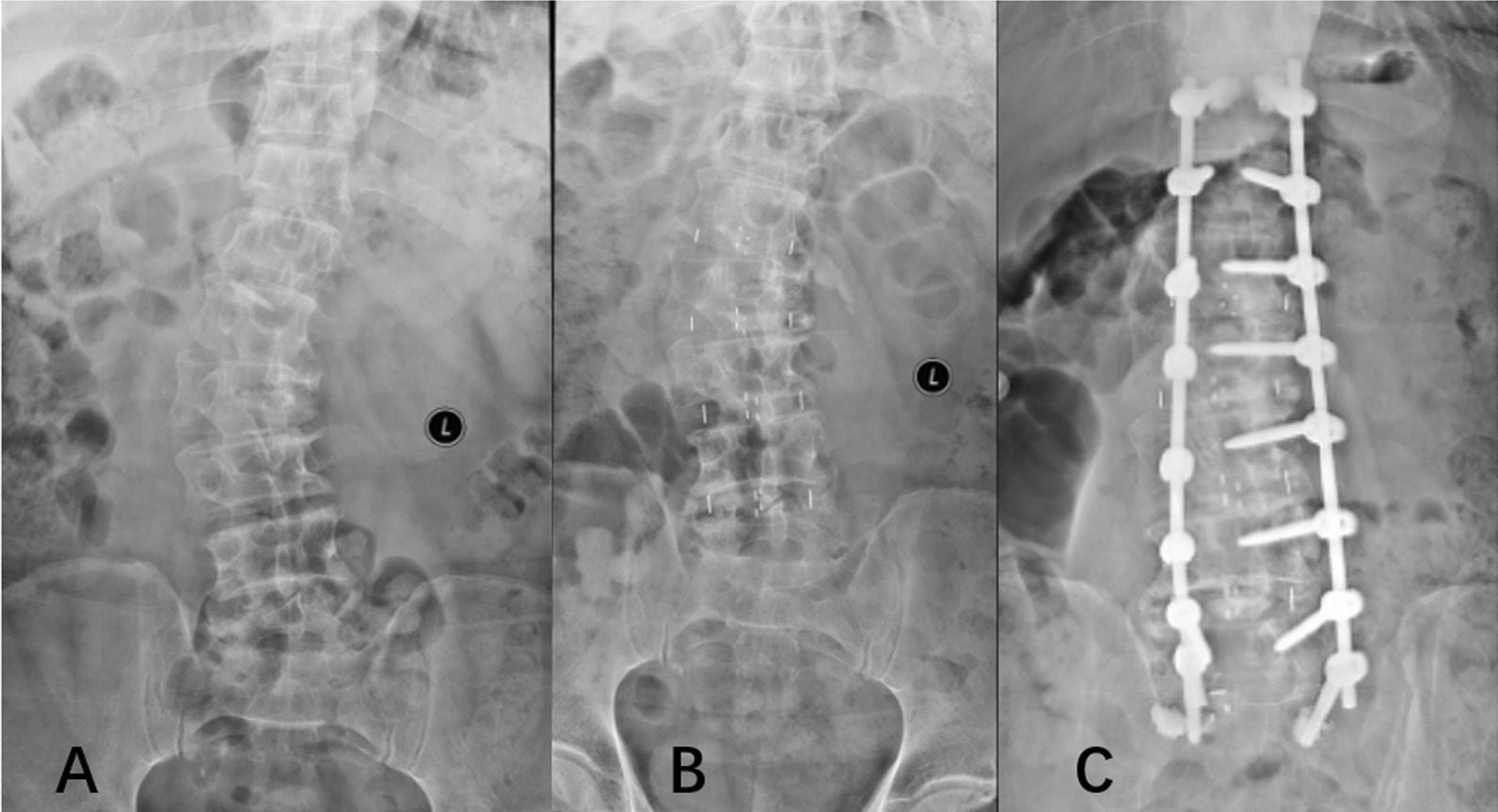
**A** is an ADS patient in the preoperative period. **B** Four wide cages opened the lateral osteophytes and reduced the curve in the first stage of surgery. **C** Posterior fixation corrected the deformity. ADS, adult degenerative scoliosis

The present study found that the number of intervertebral fusion levels was similar between the groups; however, the average volume of intraoperative blood loss was less in the OLIF group than in the PLIF group. The postoperative drainage volume was less, and the drainage time was shorter in the OLIF group than in the PLIF group. Unlike PLIF, which relies more on osteotomy correction, OLIF can achieve correction by opening the intervertebral space and lateral osteophytes. Therefore, OLIF uses fewer osteotomy levels and requires a lower grade of osteotomy than PLIF. OLIF achieved similar correction effects to PLIF by using fewer osteotomies. Furthermore, the wide cages in the OLIF group opened the lateral osteophytes, widened the intervertebral space to correct the deformity, supported the lateral rims of the endplate, and prevented subsidence and subsequent loss of deformity correction [16] (Fig. 2).

The PLIF group had more intraoperative and postoperative blood transfusion volume than the OLIF group. Six patients in the PLIF group received postoperative albumin infusion, while only three patients in the OLIF group received albumin infusion. There was no significant difference in average albumin infusion between the two groups. There was no significant difference in postoperative hemoglobin and albumin between the groups, suggesting that the nutritional statuses of the groups were similar after blood transfusion and albumin infusion. Although OLIF did not extend the surgery time, some patients require extra anesthesia for the second-stage surgery. The damage caused by second anesthesia may require further attention.

### Fixation levels

**Fig. 3:**
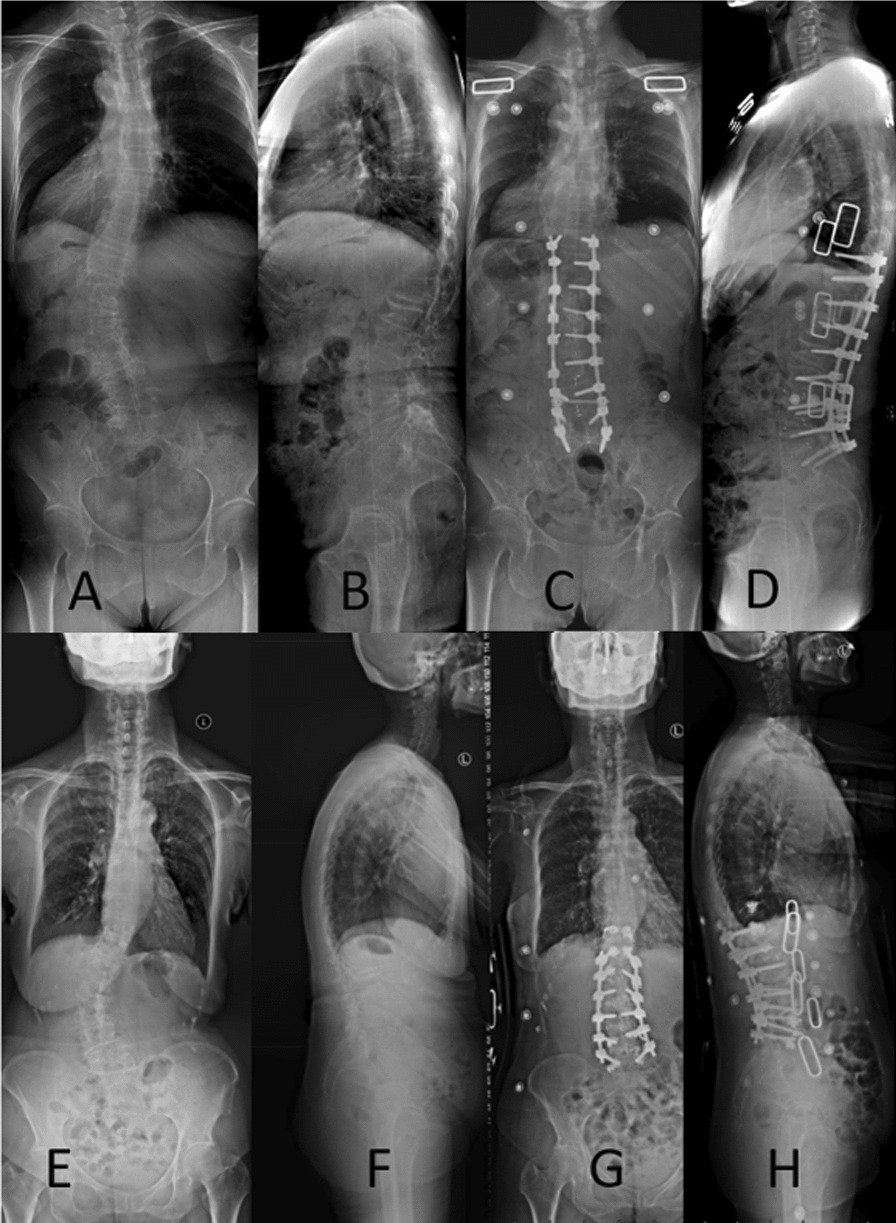
**A**–**D** is a patient in the PLIF group. **E–H** is a patient in the OLIF group. The preoperative CA of both groups was around 50°. The postoperative CA of both groups is around 15°. The OLIF group had smaller surgical trauma and shorter segments. PLIF, posterior lumbar interbody fusion; OLIF, oblique lateral interbody fusion; CA, Cobb angle

The recognizable criteria to determine fusion level are fixation, including the apex of the curve, junctional kyphosis, severe lateral subluxation, spondylolisthesis, and retrolisthesis [2]. To shorten the fusion segment, reducing the CA or downgrading the curve's apex in the first stage of OLIF is necessary. Wide cages can make the vertebra horizontal. Thus, the OLIF group had fewer fixation levels than the PLIF group (Fig. 3).

### Complications

Surgical complications include perioperative and long-term complications. Due to different surgical approaches, perioperative complications of OLIF include vascular injury, endplate destruction, transient thigh pain or numbness and hip flexor weakness or pain, ileus injury, neurologic injury, and sympathetic chain injury [13, 25]. OLIF achieves neurolysis through indirect decompression, so the probability of dural tear may be lower [26]. Osteoporosis was the main pathological basis for intraoperative endplate injury and postoperative cage subsidence. The higher fusion rate of OLIF may improve outcomes for endplate injury, cage subsidence, and other implant-related complications [13]. Long-term complications, such as pseudarthrosis and adjacent segment degeneration, are rare in OLIF group [13, 15]. Transient psoas paresis was the most common minor complication in OLIF group, whereas urinary tract infection was the most common in the PLIF group. Overall, OLIF is safe and effective in treating ADS.

### Limitations

This study was retrospective. We summarized patients who had undergone surgery rather than a prospective analysis of patients with scoliosis. Nevertheless, this study matched the age, gender, PI, and CA of the groups to minimize selection bias as much as possible. A large sample with long-term follow-up is more suitable to validate the efficacy of OLIF. Nevertheless, we believe that our results are helpful for surgeons who treat ADS.

## Conclusion

OLIF with posterior fixation surgery can achieve the same corrective effect and clinical efficacy as a posterior internal fusion with fixation surgery for treating ADS. OLIF with posterior fixation surgery introduces less trauma and reduces the number of fixation segments.

## Data Availability

The underlying data supporting the results of this study could be obtained by contacting the corresponding author.

## References

[1] Silva FE, Lenke LG (2010). Adult degenerative scoliosis: evaluation and management. Neurosurg Focus.

[2] Cho K, Kim Y, Shin S, Suk S (2014). Surgical treatment of adult degenerative scoliosis. Asian Spine J.

[3] Glassman SD, Bridwell K, Dimar JR, Horton W, Berven S, Schwab F (2005). The impact of positive sagittal balance in adult spinal deformity. Spine.

[4] Allain J, Dufour T (2020). Anterior lumbar fusion techniques: ALIF, OLIF, DLIF, LLIF, IXLIF. Orthop Traumatol Surg Res.

[5] Yamato Y, Hasegawa T, Yoshida G, Banno T, Oe S, Arima H, Mihara Y, Ide K, Watanabe Y, Kurosu K, Nakai K, Matsuyama Y (2023). The use of lateral lumbar interbody fusion for identifying adult patients with spinal deformities treatable by short corrective fusion in 2-stage surgery. J Orthop Sci..

[6] Matsukura Y, Yoshii T, Morishita S, Sakai K, Hirai T, Yuasa M, Inose H, Kawabata A, Utagawa K, Hashimoto J, Tomori M, Torigoe I, Yamada T, Kusano K, Otani K, Sumiya S, Numano F, Fukushima K, Tomizawa S, Egawa S, Arai Y, Shido S, Okawa A (2021). Comparison of lateral lumbar interbody fusion and posterior lumbar interbody fusion as corrective surgery for patients with adult spinal deformity—a propensity score matching analysis. J Clin Med.

[7] Berjano P, Lamartina C (2013). Far lateral approaches (XLIF) in adult scoliosis. Eur Spine J.

[8] Shimizu T, Fujibayashi S, Otsuki B, Murata K, Matsuda S (2021). Indirect decompression via oblique lateral interbody fusion for severe degenerative lumbar spinal stenosis: a comparative study with direct decompression transforaminal/posterior lumbar interbody fusion. Spine J.

[9] Fujibayashi S, Hynes RA, Otsuki B, Kimura H, Takemoto M, Matsuda S (2015). Effect of indirect neural decompression through oblique lateral interbody fusion for degenerative lumbar disease. Spine.

[10] Sato J, Ohtori S, Orita S, Yamauchi K, Eguchi Y, Ochiai N, Kuniyoshi K, Aoki Y, Nakamura J, Miyagi M (2017). Radiographic evaluation of indirect decompression of mini-open anterior retroperitoneal lumbar interbody fusion: oblique lateral interbody fusion for degenerated lumbar spondylolisthesis. Eur Spine J.

[11] Zhang Y, Liu C, Ge X (2022). Clinical and radiographic outcomes of stand-alone oblique lateral interbody fusion in the treatment of adult degenerative scoliosis: a retrospective observational study. BMC Musculoskelet Dis.

[12] Lee JS, Son DW, Lee SH, Sung SK, Lee SW, Song GS, Kim YH, Choi CH (2022). Surgical outcome of minimal invasive oblique lateral interbody fusion with percutaneous pedicle screw fixation in the treatment of adult degenerative scoliosis. Medicine.

[13] Zhu L, Wang J, Zhang L, Feng X (2022). Outcomes of oblique lateral interbody fusion for adult spinal deformity: a systematic review and meta-analysis. Glob Spine J.

[14] Faldini C, Di Martino A, De Fine M, Miscione MT, Calamelli C, Mazzotti A, Perna F (2013). Current classification systems for adult degenerative scoliosis. Musculoskelet Surg.

[15] Mittal S, Sudhakar PV, Ahuja K, Ifthekar S, Yadav G, Sinha S, Goyal N, Verma V, Sarkar B, Kandwal P (2023). Deformity correction with interbody fusion using lateral versus posterior approach in adult degenerative scoliosis: a systematic review and observational meta-analysis. Asian Spine J.

[16] Jo D, Seo E (2021). Efficacy and radiographic analysis of oblique lumbar interbody fusion in treating adult spinal deformity. PLoS ONE.

[17] Zhao L, Xie T, Wang X, Yang Z, Pu X, Lu Y, Song Y, Zeng J (2022). Comparing the medium-term outcomes of lumbar interbody fusion via transforaminal and oblique approach in treating lumbar degenerative disc diseases. Spine J.

[18] Li X, Wang Y, Yang K, Liu C, Zhu W, Kong C, Lu S (2023). Four types of global spine sagittal alignment and compensation mechanism in adult patients with lumbar degenerative disease. J Orthop Sci..

[19] Wang Y, Li X, Zhu W, Liu C, Kong C, Lu S (2023). Compensatory classification in spine sagittal malalignment with lumbar degeneration. BMC Musculoskelet Dis.

[20] Zhu HF, Fang XQ, Zhao FD, Zhang JF, Zhao X, Hu ZJ, Fan SW (2022). Comparison of oblique lateral interbody fusion (OLIF) and minimally invasive transforaminal lumbar interbody fusion (MI-TLIF) for treatment of lumbar degeneration disease: a prospective cohort study. Spine.

[21] Sheng SR, Geng YB, Zhou KL, Wu AM, Wang XY, Ni WF (2020). Minimally invasive surgery for degenerative spondylolisthesis: transforaminal or oblique lumbar interbody fusion. J Comp Eff Res.

[22] Lin GX, Akbary K, Kotheeranurak V, Quillo-Olvera J, Jo HJ, Yang XW, Mahatthanatrakul A, Kim JS (2018). Clinical and radiologic outcomes of direct versus indirect decompression with lumbar interbody fusion: a matched-pair comparison analysis. World Neurosurg.

[23] Hiyama A, Sakai D, Katoh H, Sato M, Watanabe M (2023). Postoperative radiological improvement after staged surgery using lateral lumbar interbody fusion for preoperative coronal malalignment in patients with adult spinal deformity. J Clin Med.

[24] Hiyama A, Katoh H, Sakai D, Sato M, Tanaka M, Nukaga T, Watanabe M (2019). Changes in spinal alignment following extreme lateral interbody fusion alone in patients with adult spinal deformity using computed tomography. Sci Rep.

[25] Kim BS, Han MS, Lee TK, Kim JY, Lee JK, Moon BJ (2022). What clinicians should consider when performing oblique lumbar interbody fusion in a patient with long vertebral body osteophytes. World Neurosurg.

[26] Jin C, Xie M, He L, Xu W, Han W, Liang W, Qian Y (2019). Oblique lumbar interbody fusion for adjacent segment disease after posterior lumbar fusion: a case-controlled study. J Orthop Surg Res.

